# Genistein-3′-sodium sulfonate Attenuates Neuroinflammation in Stroke Rats by Down-Regulating Microglial M1 Polarization through α7nAChR-NF-κB Signaling Pathway

**DOI:** 10.7150/ijbs.56800

**Published:** 2021-03-08

**Authors:** Chaoming Liu, Song Liu, Lijiao Xiong, Limei Zhang, Xiao Li, Xingling Cao, Jinhua Xue, Liangdong Li, Cheng Huang, Zhihua Huang

**Affiliations:** 1 Key Laboratory of Prevention and treatment of cardiovascular and cerebrovascular diseases of Ministry of Education, Gannan Medical University, Ganzhou 341000, China.; 2 Department of Physiology, School of Basic Medical Sciences, Gannan Medical University, Ganzhou 341000, China.; 3 First Affiliated Hospital of Gannan Medical University, Ganzhou 341000, China.; 4 Department of Pathobiology, JiangXi College of Traditional Chinese Medicine, Fuzhou, 344000, China.

**Keywords:** Microglia polarization, Neuroinflammation, Ischemic stroke, α7nAChR-NF-κB signaling, Genistein-3′-sodium sulfonate.

## Abstract

Microglial M1 depolarization mediated prolonged inflammation contributing to brain injury in ischemic stroke. Our previous study revealed that Genistein-3′-sodium sulfonate (GSS) exerted neuroprotective effects in ischemic stroke. This study aimed to explore whether GSS protected against brain injury in ischemic stroke by regulating microglial M1 depolarization and its underlying mechanisms. We established transient middle cerebral artery occlusion and reperfusion (tMCAO) model in rats and used lipopolysaccharide (LPS)-stimulated BV2 microglial cells as *in vitro* model. Our results showed that GSS treatment significantly reduced the brain infarcted volume and improved the neurological function in tMCAO rats. Meanwhile, GSS treatment also dramatically reduced microglia M1 depolarization and IL-1β level, reversed α7nAChR expression, and inhibited the activation of NF-κB signaling in the ischemic penumbra brain regions. These effects of GSS were further verified in LPS-induced M1 depolarization of BV2 cells. Furthermore, pretreatment of α7nAChR inhibitor (α-BTX) significantly restrained the neuroprotective effect of GSS treatment in tMCAO rats. α-BTX also blunted the regulating effects of GSS on neuroinflammation, M1 depolarization and NF-κB signaling activation. This study demonstrates that GSS protects against brain injury in ischemic stroke by reducing microglia M1 depolarization to suppress neuroinflammation in peri-infarcted brain regions through upregulating α7nAChR and thereby inhibition of NF-κB signaling. Our findings uncover a potential molecular mechanism for GSS treatment in ischemic stroke.

## Introduction

Stroke is the third leading cause of death among human diseases, and over 80% of stroke results from brain ischemia 1, 2. Currently, therapeutic strategies for ischemic stroke are limited: administration of tissue plasminogen activator (tPA) or mechanical thrombectomy to gain brain reperfusion. However, the therapeutic window of these approaches is exceptionally narrow: generally less than 8 h 1, 3-5. Thus, seeking novel drugs or other therapeutic strategies are still the concerns of neuroscientists.

Ischemic stroke activates inflammatory cascades in the acute phase, resulting in increased production of pro-inflammatory cytokines and chemokines, aggregation and adhesion of inflammatory cells, impairing of the blood-brain barrier (BBB), and subsequently exacerbating brain damage 6, 7. Post-stroke neuroinflammation is considered as a favorable target for the treatment of ischemic stroke 1, 3, 7, 8. Microglia, one of the essential participants in neuroinflammation, is activated in a few minutes of ischemic stroke hitting 9, 10. Activated microglial cells polarize to M2 phenotype in the early stage of ischemic stroke and subsequently switch to M1 phenotype 11. The roles of M2 and M1 polarization of microglia in neuroinflammation are antagonistic: M2 phenotype microglial cells exert anti-inflammatory and neuroprotective effects by secreting transforming growth factor β (TGF-β) and interleukin-10 (IL-10), while M1 polarization produces pro-inflammatory cytokines such as interleukin-1β (IL-1 β), IL-6 and tumor necrosis factor α (TNF-α), which contribute to the disruption of BBB and the deterioration of brain injury 3, 12, 13. Thus, reducing microglial M1 depolarization is a critical strategy to limit brain's inflammatory damage in ischemic stroke 14-18.

Cholinergic anti-inflammatory pathway (CAIP) is mediated by the stimulation of vagal nerve, which releases acetylcholine (ACh) to modulate cerebral inflammation mainly through α7 nicotinic acetylcholine receptor (α7nAChR) 14, 19, 20. This receptor is expressed in the membrane of microglia, neurons, and endothelial cells in the brain. Activation of α7nAChR reduces microglial activation, inhibits the production of pro-inflammatory cytokines, and attenuates brain injury and functional deficits 6, 21, 22. Moreover, it was reported that activation of α7nAChR inhibited nuclear factor kappa B (NF-κB) pathway including blocking the phosphorylation of IκB and thereby restraining the nucleus translocation of p65-NF- κB, a subunit of NF-κB 23, 24. In ischemic stroke mice, α7nAChR agonist reduced the brain injury through decreasing the phosphorylation of p65-NF-κB protein in microglia 25-27, demonstrating that α7nAChR-mediated inhibition of NF-κB signaling is a crucial neuroprotective mechanism against ischemic brain injury.

Estrogen displays potent neuroprotective effects, whereas long-term estrogen treatment increases the incidence risk of breast cancer 28-30. Therefore, phytoestrogens are seeing as an alternative to estrogen. Genistein is widely studied as a phytoestrogen which protects against ischemic stroke-induced brain injury. However, the bioavailability of genistein is limited because of its poor water and lipid solubility 31. Genistein-3'-sodium sulfonate (GSS) is a sulfonated product of genistein, which has a better water solubility than genistein. Our previous research indicated that GSS treatment significantly alleviated cerebral edema and brain infarction in transient middle cerebral artery occlusion (tMCAO) rats 32. Besides, we also found that Genistein has a therapeutic effect in tMCAO rat because it down-regulates inflammatory cytokines levels such as IL-1β, TNF-α and IL-6, demonstrating that GSS precursor has significant anti-inflammation effects 33. 17β-Estradiol and progesterone promote microglial depolarization from M1 to M2 phenotype in ischemic stroke 34, 35. Therefore, we hypothesized that GSS could inhibit microglial M1 depolarization through regulating α7nAChR-mediated inhibition of NF-κB pathway and thus suppress the neuroinflammation and protect against the brain injury in ischemic stroke. In this study, we investigated the effects of GSS on microglial M1 polarization and its underlying molecular mechanisms using tMCAO rats and lipopolysaccharide (LPS)-treated microglial cells.

## Materials and methods

### Animal and Drugs

Animal experiments were performed strictly following the guidelines of the Animal Care and Use Committee of Gannan Medical University. Sprague-Dawley (SD) rats (Male, 250 g) were purchased from Hunan SJA Laboratory Anima Company (Hunan, China) and were housed at 50-60% relative humidity and a temperature of 22-25℃. The rats drank sterile running water and took SPF grade chow freely. Before performing experiments, all rats were acclimatized to the laboratory environment for at least one week.

GSS was purchased from Shanghai Skychemical Co., Ltd (Shanghai, China), and the chemical structure of it was showed in our preview study 32. It was dissolved in normal saline (NS) to 1 mg/mL concentration before use. Specific α7nAChR-antagonist α-bungarotoxin (α-BTX, #ab120542) was obtained from Abcam (Cambridge, United Kingdom). It was dissolved in 0.1 M phosphate buffer solution (PBS).

### Reagents

Tris, sodium dodecyl sulfate (SDS), 30% acrylamide and protein lysis buffer (#R0010) were obtained from Solarbio Science & Technology Company (Beijing, China). TRzol was purchased from Ambion company (Texas, United States). Anti-CD11b (#ab1211, #ab133357), anti-IL-1β (#ab9787), anti-CD40 (#ab13545), anti-CD68 (#ab125212), and anti-α7nAChR (#ab10096) were purchased from Abcam (Cambridge, United Kingdom). Anti-IKK (#2682s), anti-phospho-IKK(#2697s), anti-P65-NF-κB (#242s), anti-phospho-P65--NF-κB (#3033s), anti-IκB (#4814s) and anti-phospho-IκB (#9246s) were purchased from Cell Signaling Technology (Massachusetts, United States). Anti-β-tubulin (#MA511732), AlexaFluor488 mouse secondary antibody (#A11029), AlexaFluor488 rabbit secondary antibody (#A11034), enhanced chemiluminescence reagent (ECL) Western blotting detection reagents (#32106), Reverse Transcription Master Mix kit (Invitrogen, #4374966), SYBR^®^ Select Master Mix (Life Technologies, #4472908), 0.25% trypsin (Gibco, #25200-056), DMEM medium (Gibco, #31800-014), fetal bovine serum (FBS) (Gibco, #10099-141) and penicillin streptomycin combination (Gibco, #15140-122) were purchased from Thermo Fisher Scientific (Massachusetts, United States). 2-, 3-, 5-triphenyltetrazolium chloride (TTC, #T8877-25G) was purchased from Sigma (United States).

### Transient middle cerebral artery occlusion and reperfusion (tMCAO) rat model and drug treatments

Rat tMCAO model was established, as described previously 32, 36. Briefly, rats were anesthetized with 1% pentobarbital sodium (50 mg/kg, i.p.), and brain ischemia was induced by inserting a 5-cm-long nylon filament (diameter, 0.24-0.28 mm) into the middle cerebral artery for 2 h. Then, the filament was removed to allow reperfusion of the brain for 24 h. Sham rats were performed a comparable surgery as tMCAO rats but without the occlusion of the middle cerebral artery. GSS (1.0 mg/kg) was administrated via sublingual vein injection 10 min after ischemia. Since α-BTX is not able to pass through the blood-brain barrier, we injected it into the lateral ventricle at a dose of 0.5 µg/kg body weight 30 min before tMCAO surgery. Rats without α-BTX and GSS treatments were parallelly given equivalent vehicle (saline). The schematic diagram of animal treatments is shown in **Fig S1A** and **B** in supplementary materials. The doses of GSS and α-BTX were chosen based on previous study and our preliminary experiments 32.

### Lateral ventricular injection

Rats were anesthetized with 1% pentobarbital sodium (50 mg/kg, i.p.) and fixed on a stereotactic apparatus (ZH-1-Lanxing, RWD, China). When bregma was exposed, a burr hole was prepared on the skull in the right hemisphere (1.6 mm lateral and 0.9 mm posterior to bregma) using a power drill (68605, ϕ 1.4 mm, RWD, China). Then an injector (5-μL micro-syringe, Hamilton, USA) connected with micro-pump (KDS310, Harvard Apparatus, USA) was placed its needle into the right lateral ventricle at 3.5 mm depth of sub-dural through the micro-hole. Placement of needle was performed using a stereotactic apparatus. The vehicle or the α-BTX was then administered into the lateral brain ventricle at a speed of 0.25 μL/min. The needle stayed in place for 5 min after injection to prevent backflow.

### Neurological test

All rats were received Garcia assessment at 24 h after cerebral ischemia reperfusion (I/R) to evaluate neurobiological function. The evaluation includes six tests scoring on a scale from 3 to 18 37: spontaneous activity, symmetry in four limb movements, forepaw outstretching, climbing, body proprioception, and response to vibrissae touch. Repeat each test 3 times and take the average as an evaluation score.

### TTC staining

After the neurological assessment, rats were anesthetized with 1% pentobarbital sodium (50 mg/kg, i.p.) and sacrificed. Rat brain was collected and performed 2,3,5-Triphenyltetrazolium chloride (TTC) staining. In brief, rat brain was consecutively sliced into six coronal sections (2 mm thickness) and then stained with 0.5% TTC solution for 30 min at 37℃ in a water bath shaker in the dark. During the staining, the slices were turned over every 5 min. After the staining, the slices were washed with PBS and fixed in 4% paraformaldehyde for 6 h. The infarcted area and brain area in TTC-stained brain slices were measured using ImageJ 1.37a. The corrected percentage of infarct volume was calculated as the formula: infarct volume (%) = (the area of contralateral hemisphere - the area of non-infarcted ipsilateral hemisphere) /(2 * the area of contralateral hemisphere) *100% 37.

### Cell culture and treatments

BV2 microglial cells were purchased from the Cell Bank of Type Culture Collection of Chinese Academy of Sciences (Shanghai, China). BV2 cells were grown in Dulbecco's modified Eagle's medium (DMEM) containing 10% fetal bovine serum, 100 U/mL penicillin, and 100 μg/mL streptomycin in a cell incubator with 5.0% CO_2_ at 37°C. Cells were seeded in culture dishes at a density of 1×10^6^ cells/mL and treated with LPS (1.0 μg/mL) for 24 h to induce M1 polarization. A schematic diagram for *in vitro* experiments is shown in figure S1 C and D in supplementary materials. These cell experiments were performed at least three independent times with at least three biological replicates.

To determine the effects of GSS on M1 polarization, cells were randomly allocated into four groups: control group (treated with vehicle), LPS group (treated with LPS), LPS+GSS group (treated with LPS plus GSS treatment, 10 μM), and GSS group (treated with GSS alone). Cells were treated with GSS for 24 h.

In order to determine whether α7nAChR-mediated NF-κB signaling was involved in the effects of GSS on microglial M1 polarization, cells were randomly divided into six groups: control group (treated with vehicle), LPS group (treated with LPS), LPS+GSS group (treated with LPS plus GSS treatment, 10 μM), GSS group (treated with GSS alone), α-BTX+LPS+GSS group (pretreated with α-BTX followed by LPS and GSS treatment), and α-BTX+LPS group (pretreated with α-BTX followed by LPS incubation). Cells were pretreated α-BTX (10 nM) 30 min before given LPS incubation.

### Western blot

Ischemic penumbra brain tissues were collected for western blotting. Brain tissues were added protein lysis buffer and homogenized on ice for 1 h. BV2 cells were also lysed with protein lysis buffer on ice for 1 h. Then these samples were centrifuged at 12,000 g for 15 min to obtain the supernatants. The protein concentration in samples was measured by BCA kit according to the manufacturer's instructions. Equivalent total protein (30 μg) in each sample was separated in SDS/PAGE gel by electrophoresis and transferred to PVDF membrane. After blocking with 5% non-fat milk, the membrane was incubated with following primary antibodies overnight at 4℃: CD11b (1:1000), IL-1β (1: 500), CD40 (1:500), CD68 (1:500), α7nAChR (1:500), IKK (1:1000), phospho-IKK (1:500), P65-NF-κB (1:1000), phospho-p65-NF-κB (1:500), IκB (1:1000), phospho-IκB (1:500) and β-tubulin (1:2000). Then the membrane was incubated with respective secondary antibodies (1:5000) at room temperature for 1 h and then incubated with an enhanced chemiluminescence reagent for 1 min. Finally, protein band images were captured, and the gray-scale of each band was analyzed using Chemiluminescence Imaging System (Amersham™ Imager 600, United States). All experiments were run in triplicate and repeated a minimum of three times independently with consistent results.

### Quantitative PCR (qPCR)

Total RNA was isolated from tissues or cells using TRIzol reagent according to the manufacturer's instructions. 4 μg total RNA was used to synthesize the first strand cDNA using Reverse Transcription Master Mix kit (SYBR^®^ Select Master Mix). Then PCR reaction was run in triplicate and performed as following reaction system: cDNA 2 μL, 1x SYBRÒ 10 μL, 10 μM primer 2 μL, adding ddH_2_O to a total volume of 20 μL. Parameters for PCR reaction were as bellows: pre-denatured at 95℃ for 20 min follow by 40 cycles (desaturated at 95 ℃ for 10 s, annealed at 61 ℃ for 20 s, and extended at 72 ℃ for 25 s). PCR results were normalized to GAPDH and expressed as folds of sham or control group. The sequence of primers used in this study was shown in Table 1.

### ELISA assay

The concentration of IL-1β in the culture supernatant was determined using enzyme-linked immunosorbent assay (ELISA) kit (R&D systems, Minneapolis, MN) according to the manufacturer's instructions.

### Immunofluorescence

Rats were anesthetized and fixed the whole animal with 4% paraformaldehyde through transcranial perfusion. The brain was then taken out, immersed in 4% paraformaldehyde, dehydrated with 10% sucrose, and cut into brain slices (30 μm thickness) by freezing microtome. Dried brain slices were hydrated with PBS at room temperature (RT) for 20 min, and then the slices were incubated in 0.3 % Triton X-100/PBS solution at RT for 10-15 min. After washed with PBS for 5 min×3 times, the slices were blocked with 3% BSA at RT for 30 min and incubated with anti-CD11b antibody (1:200) or α7nAChR (1:200) at 4℃ overnight. Then the slices were washed with PBS and incubated with AlexaFluor488 mouse secondary antibody (1:2000). Finally, the slices were stained with DAPI and sealed with 50% glycerin. The immunofluorescent images were captured under a fluorescence microscope (Carl Zeiss Lsm880, Germany).

Immunofluorescence for BV2 cells was performed as bellows: cells were fixed with cold methanol at -20℃ for 5 min, washed with PBS for 5 min×3 times. The subsequent staining procedure was the same as above brain slice immunofluorescence, except for incubating with anti-IL-1β (1:100) or anti-P65-NF-κB (1:100), and then incubated with AlexaFluor488 rabbit secondary antibody (1:2000).

### Statistical analysis

Data were presented with mean ± standard deviation (SD). Statistical analysis was performed using SPSS 20.0 software. One-way ANOVA with Newman-Keuls test was used to compare the differences between the means of more than two groups. The value of *P*<0.05 was considered statistically significant.

## Results

### GSS treatment reduced brain injury and neurological deficit in tMCAO rats

**Figure 1:**
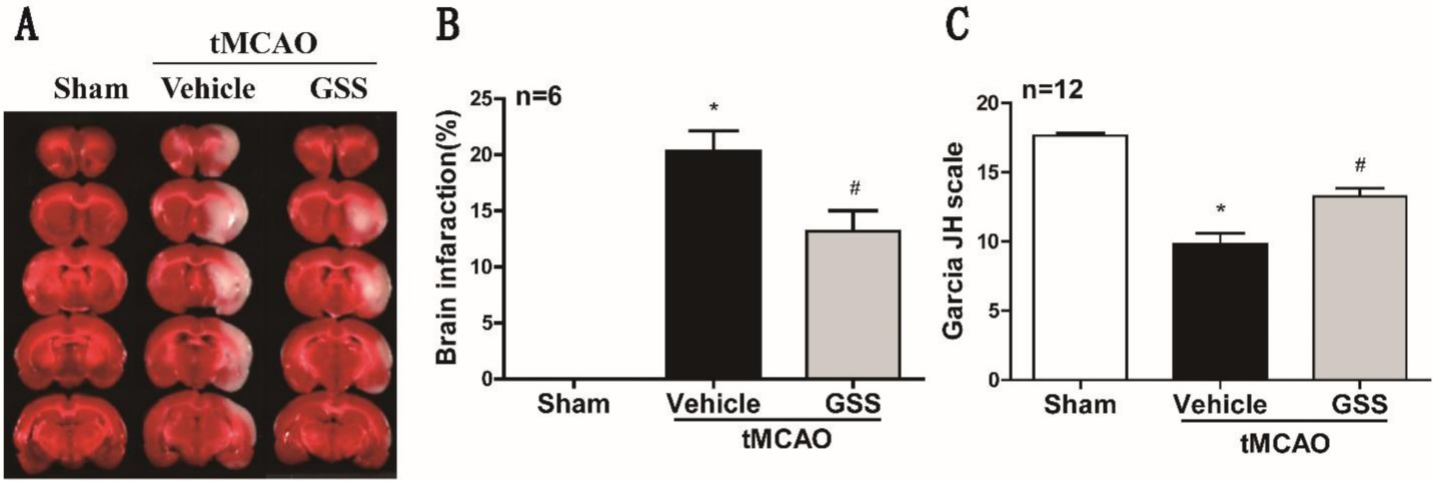
** GSS treatment reduced brain infarct volume and improved neurologic function in tMCAO rats.** Transient MCAO rats were treated with 1.0 mg/kg GSS or equivalent vehicle as indicated, then brain infarcted volume and neurological function scores were evaluated. (A) The representative images of TTC staining for infarcted brain were shown. (B) Analysis results of brain infarcted volume. (C) Analysis results of neurological function scores. Comparisons between groups were performed using one-way ANOVA followed by Newman-Keuls test. ^*^P<0.05 versus sham group, ^#^*P*<0.05 versus tMCAO group.

TTC staining and neurological deficit evaluation results showed that vehicle-administrated tMCAO rats (tMCAO group) developed significant brain infarction and neurological deficit at 24 h after I/R compared with sham rats, which were significantly inhibited by GSS treatment (Figure 1).

### GSS treatment inhibited microglial M1 polarization in the brain peri-infarct area in tMCAO rats

**Figure 2:**
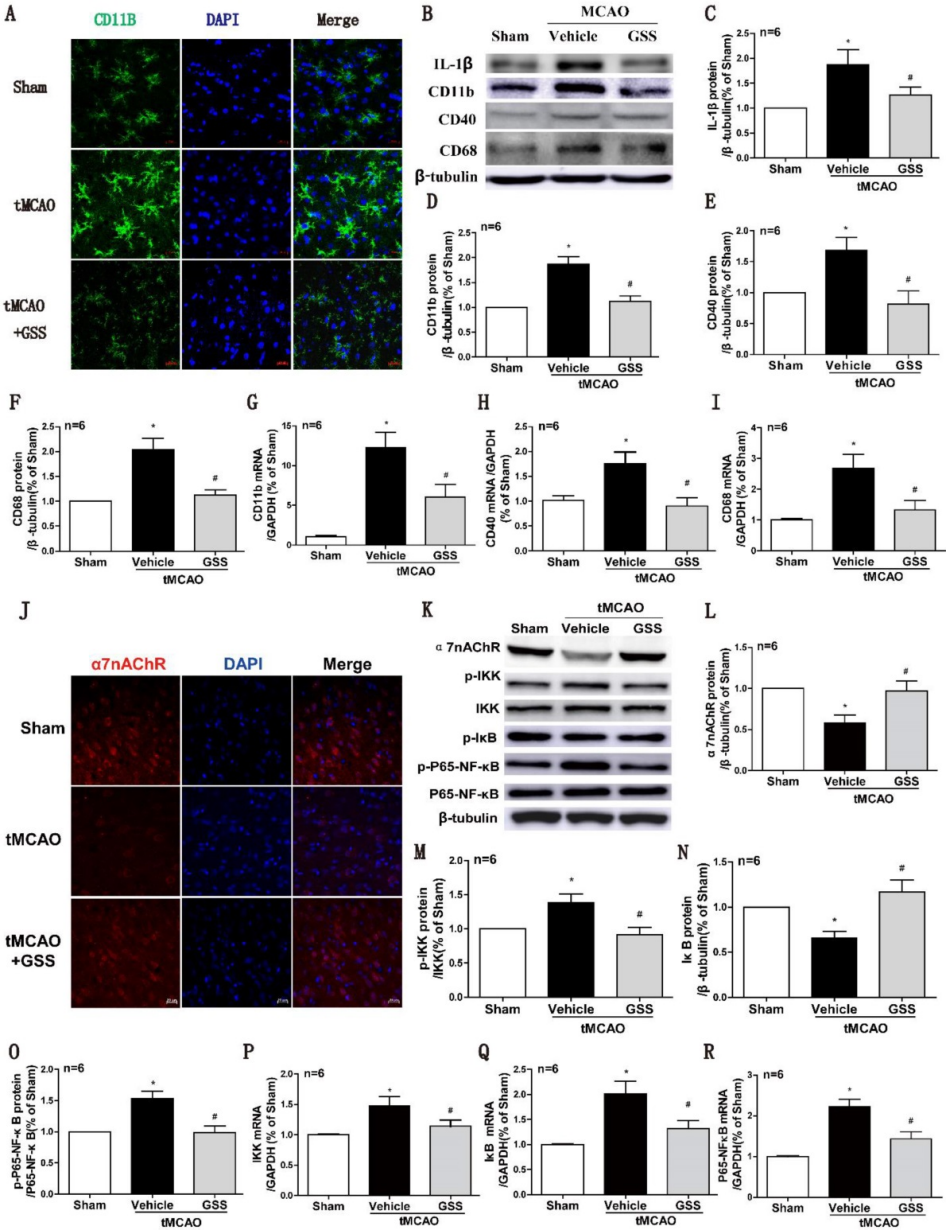
** GSS treatment inhibited microglial M1 polarization and α7nAChR-NF-κB signaling activation in tMCAO rats.** Microglial M1 depolarization and α7nAChR-NF-κB signaling activation in the ischemic penumbra region was determined 24 h I/R. (A) Microglia morphology was determined by immunofluorescent staining of CD11b (Scale bar = 20 μm, n=6). (B-F) Western results of IL-1β, CD11b, CD40 and CD68. (G-I) CD11b, CD40 and CD68 mRNA expression determined by qPCR. (J) Representative images of immunofluorescent staining of α7nAChR (Scale bar = 20 μm, n=6). (K-O) Western blot results of α7nAChR and NF-κB signaling proteins. (P-R), the qPCR results of IKK, IκB and P65-NF-κB mRNA expression. Comparisons between groups were carried out using one-way ANOVA followed by Newman-Keuls test. *P<0.05 versus sham group, #P<0.05 versus tMCAO group.

Next, we used CD11b as a cell surface marker to determine microglial activation. As shown in Fig 2A, the morphology of microglia in sham group was ramified with a small cell body, which was in a resting phenotype, whereas most microglial cells were activated in the peri-infarct area of brain of tMCAO rats, characterized by a rod-shape morphology with a larger cell body, a fusion of cell nucleus, and thick or less number of cellular processes, which were significantly inhibited by GSS treatment. In the meantime, Real-time qPCR and western blot results showed that mRNA and protein expression of M1 microglial markers (CD11b, CD40 and CD68) significantly increased in tMCAO group. Whereas, these changes were significantly attenuated by GSS treatment in tMCAO rats (Figure 2B, D-I). Besides, GSS treatment also significantly inhibited the increase of IL-1β protein expression in the peri-infarct brain region induced by tMCAO insult (Figure 2B and C).

### GSS treatment restored α7nAChR protein expression while suppressed NF-κB signaling in tMCAO rats

**Figure 3:**
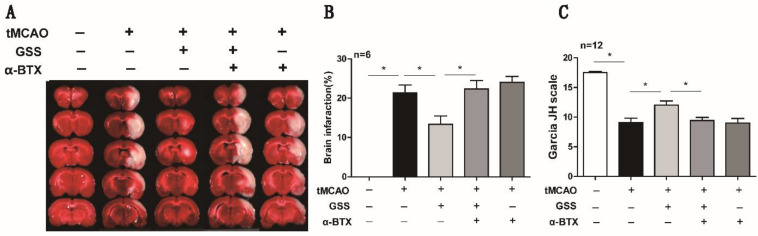
** α-BTX pretreatment reversed the therapeutic effects of GSS treatment on brain injury and neurologic function in tMCAO rats.** α-BTX (0.5 μg/kg) was administrated by stereotaxic injection 30 min before the tMCAO surgery. Brain infarct size was measured by TTC staining 24 h after I/R. (A) and (B) Representative images and analysis result of TTC staining for the evaluation of brain infarcted volume were shown, respectively. (C) Analysis result of neurological function scores. Comparisons between groups were carried out using one-way ANOVA followed by Newman-Keuls test. *P<0.05.

NF-κB signaling pathway is regulated by α7nAChR, attributing to M1 polarization of microglia 39, 40. Real-time qPCR results showed that mRNA expression of IKK, IκB and P65-NF-κB in the ischemic penumbra significantly increased in tMCAO group compared with sham group. GSS treatment significantly inhibited tMCAO-induced increases of phosphorylated IKK and phosphorylated p65 protein, but restored IκB and α7nAChR protein expression in the ischemic penumbra region (Figure 3J-O). GSS treatment significantly reduced mRNA expressions of these genes in tMCAO rats (Figure 2P-R).

### α7nAChR inhibitor blocked the protective effects of GSS against brain injury after tMCAO

**Figure 4:**
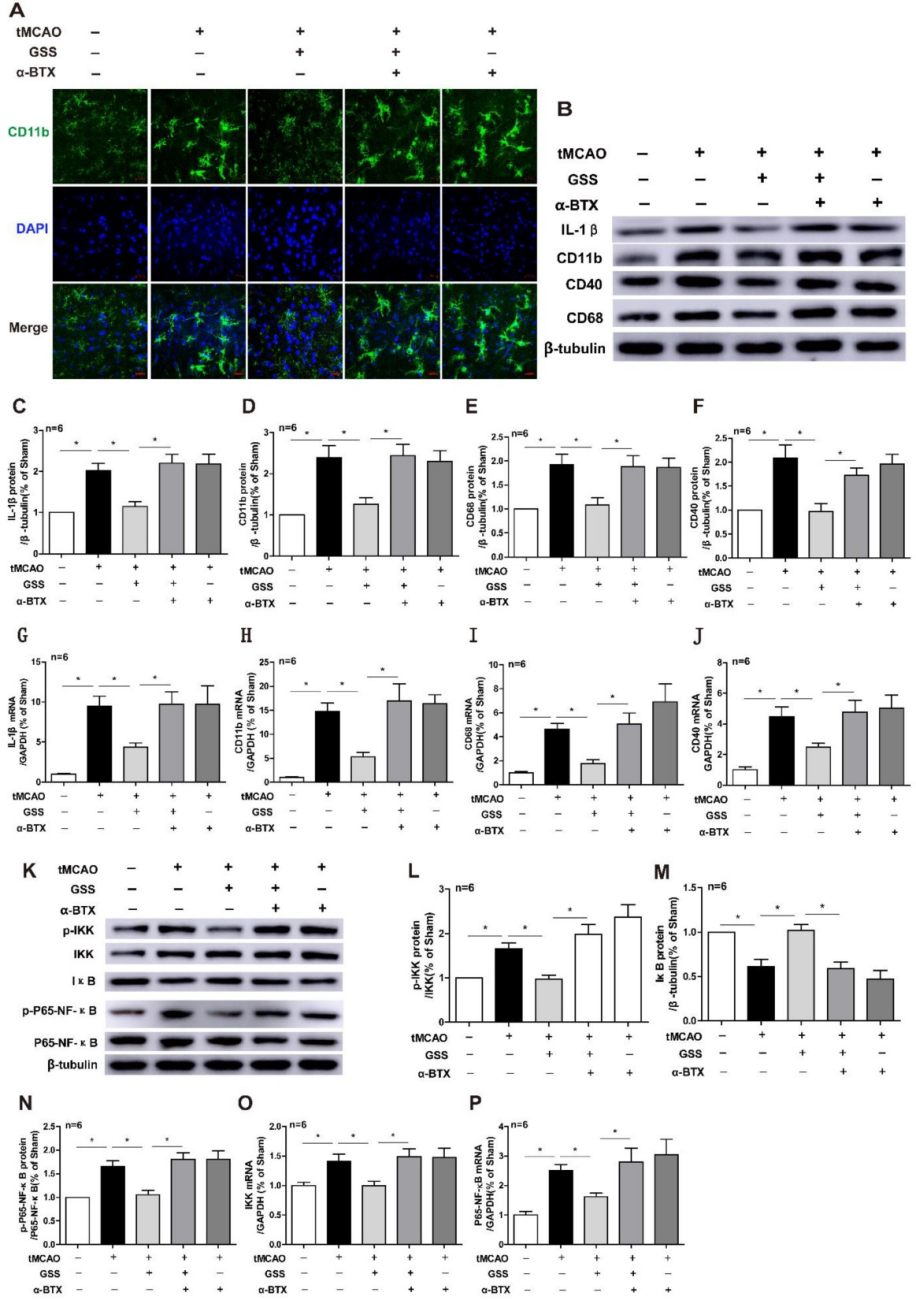
** α-BTX pretreatment inhibited the changes of microglia M1 polarization and α7nAChR-NF-κB signaling caused by GSS treatment in tMCAO rats.** α-BTX (0.5 μg/kg) was administrated by stereotaxic injection 30 min before the tMCAO surgery. Microglia M1 depolarization and expression of α7nAChR-NF-κB signaling was measured 24 h after I/R. (A) Representative images of immunofluorescent staining of CD11b (Scale bar = 20 μm, n=6). (B-F) Western blot results of IL-1β, CD11b, CD68 and CD40. (G-J) qPCR results of CD11b, IL-1β, CD68 and CD40. (K-N) Western blot results of protein expression of NF-κB pathway. (O) and (P) qPCR results of IKK and P65-NF-κB. Comparisons between groups were carried out using one-way ANOVA followed by Newman-Keuls test. *P<0.05.

Next, we applied a specific α7nAChR inhibitor, α-BTX, to illustrate whether GSS inhibited microglial M1 polarization and brain injury through activating α7nAChR signaling. We found that α-BTX pretreatment alone did not change the infarction area and neurological deficit in tMCAO rats but significantly suppressed the therapeutic effects of GSS on brain injury and neurological function (Figure 3). Pretreatment with α-BTX also reversed the effects of GSS on the morphological change of microglia (Figure 4A) as well as mRNA and protein expression of M1 microglial markers (CD11b, CD40 and CD68) and IL-1β in the ischemic penumbra of tMCAO rats (Figure 4B-J). As shown in Fig 6A and B, we observed that α-BTX pretreatment significantly blocked the inhibitive effects of GSS on NF-κB signaling to exhibit as increasing mRNA expressions of IKK and P65-NF-κB and phosphorylation of IKK and p65 while reducing IκB protein expression to a comparable level as tMCAO group (Figure 4K-P).

### GSS treatment inhibited LPS-induced M1 polarization of microglial cells

**Figure 5:**
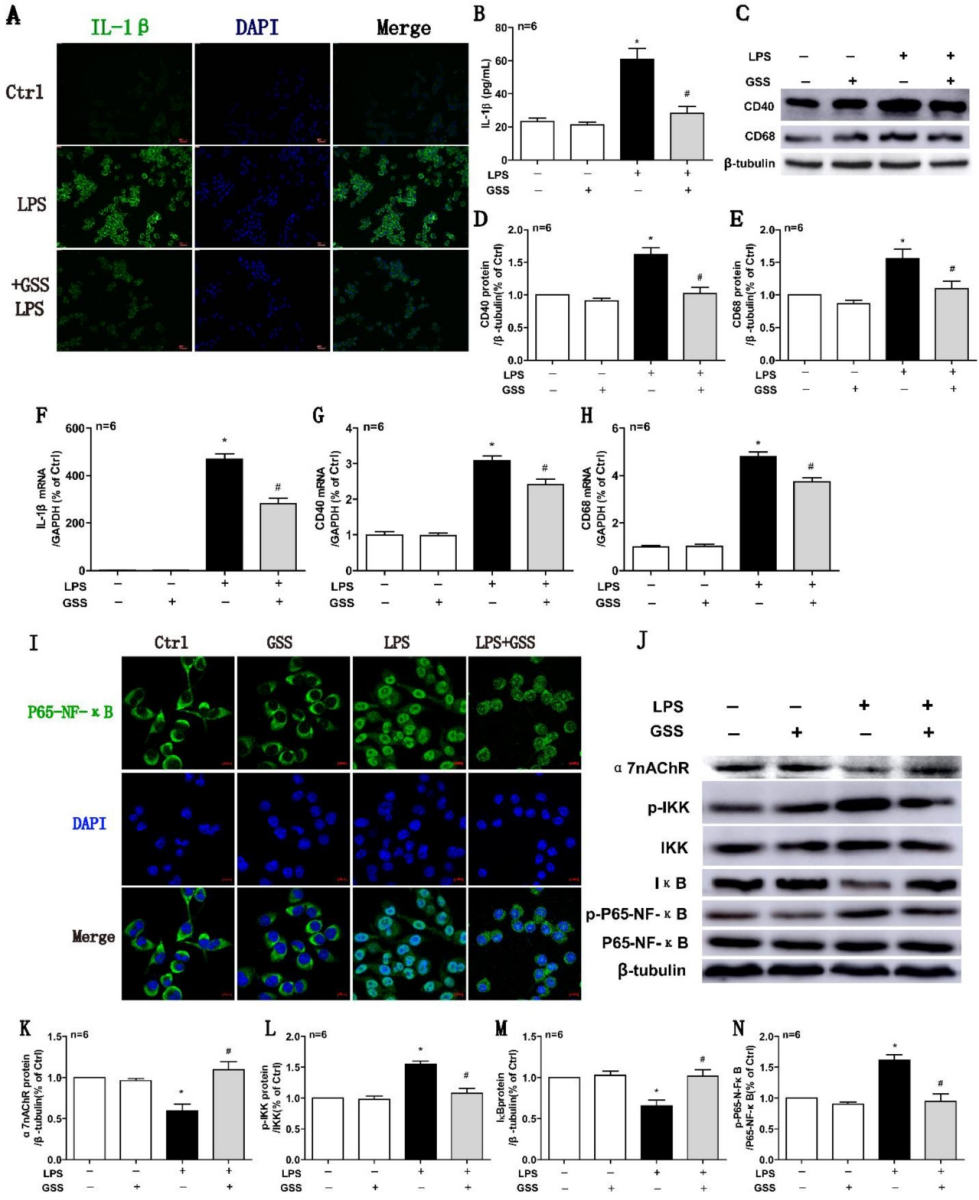
** GSS inhibited LPS-induced M1 polarization and α7nAChR-NF-κB signaling activation of BV2 microglial cells.** M1 polarization and the mRNA or protein expression of α7nAChR-NF-κB signaling of LPS-stimulated BV2 cells was determined 24 h after GSS treatment. (A) Immunofluorescence results of IL-1β (Scale bar = 500 μm, n=6). (B) ELISA result of IL-1β. (C-E) Western blot results of CD40 and CD68. (F-H) qPCR results of IL-1 β, CD40 and CD68. (I) Immunofluorescence results of P65-NF-κB (Scale bar = 10 μm, n=6). (J-N) Western blot results of α7nAChR and NF-κB signaling proteins. Comparisons between groups were carried out using one-way ANOVA followed by Newman-Keuls test. ^*^P<0.05 versus control group, ^#^*P*<0.05 versus LPS group.

We further used LPS-stimulated BV2 microglial cells to reaffirm the observed effects of GSS on M1 microglial polarization *in vivo*. ELISA results showed that LPS (1 μg/mL) stimulating for 24 h significantly upregulated the concentration of IL-1β in the cell culture supernatant compared with vehicle-treated cells. In contrast, the increase of IL-1β protein in the supernatant was significantly reduced by GSS (10 μM) treatment (Figure 5B). We also applied cell immunofluorescent staining and qPCR to validate the finding of ELISA. Consistently, the upregulation of IL-1β protein (Figure 5A) and mRNA expression (Figure 5F) in BV2 induced by LPS were significantly inhibited by GSS treatment. Besides, LPS stimulus significantly increased CD40 and CD68 mRNA expression in BV2 cells, which was significantly inhibited by GSS treatment (Figure 5G and H). Similarly, GSS treatment also significantly suppressed the increase of CD40 and CD68 protein expression induced by LPS (Figure 5C-E). GSS treatment alone did not significantly affect the expression of M1 polarization makers (IL-1β, CD40 and CD68) compared with that of control (Figure 5A-H).

### GSS treatment restored α7nAChR expression while blocked NF-κB signaling in LPS-stimulating BV2 cells

We determined the intracellular localization of P65-NF-κB using immunofluorescent staining. As shown in figure 5I, P65-NF-κB dominantly located in the cell nucleus after LPS stimulation, while it distributed mainly in the cytoplasm after GSS treatment in LPS-stimulated BV2 cells. Meanwhile, α7nAChR and IκB protein expression were significantly decreased, while expressions of p-IKK and p-P65-NF-κB were significantly increased in LPS-treated BV2 cells compared with vehicle-treated cells, which were significantly blocked by GSS treatment (Figure 5J-N). GSS treatment alone did not significantly affect α7nAChR expression and NF-κB signaling compared with that of control (Figure 5I-N).

### α7nAChR blocker antagonized the protective effects of GSS against microglial M1 polarization and NF-κB signaling

**Figure 6:**
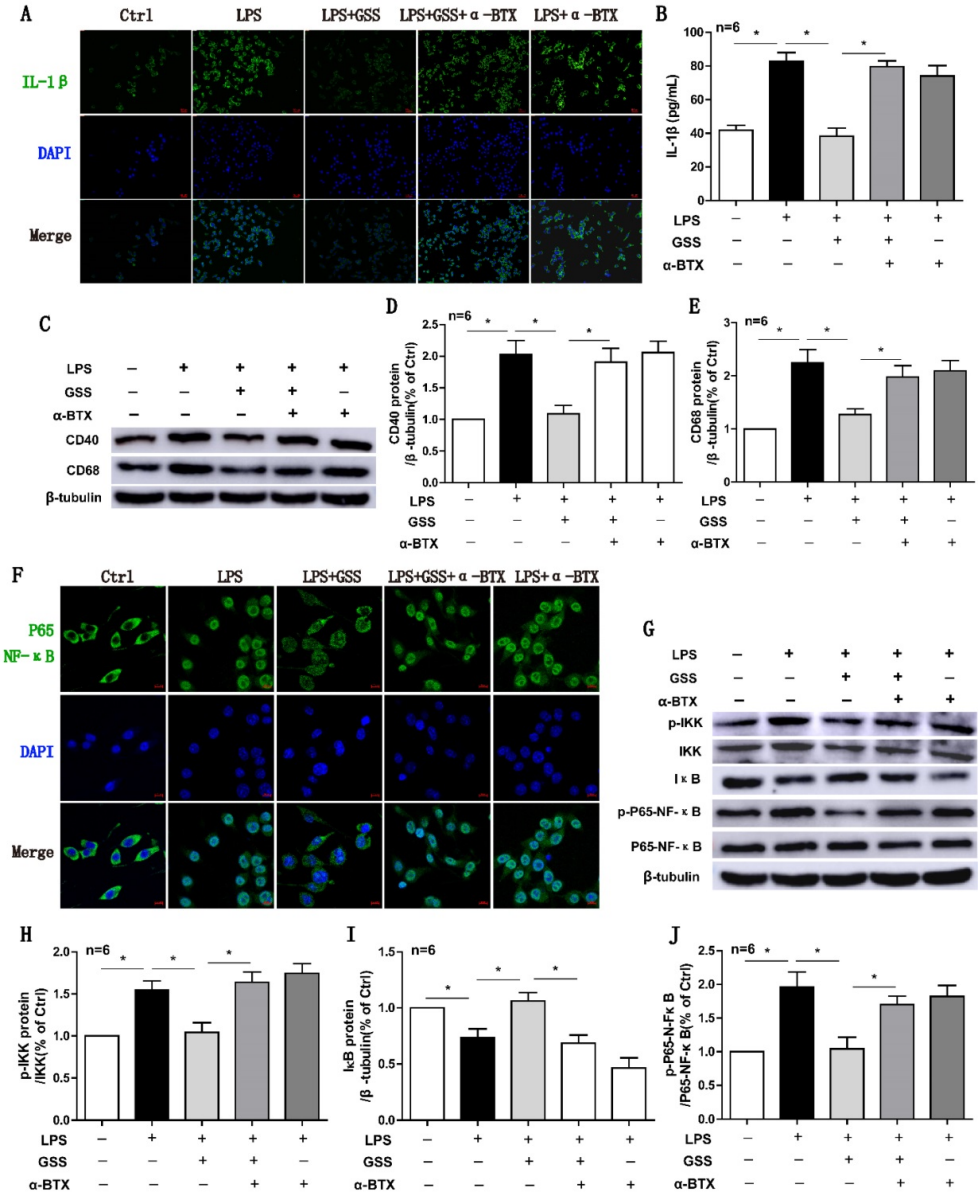
**α7nAChR inhibitor reversed the effects of GSS on M1 polarization and NF-κB signaling in LPS-stimulated BV2 cells.** Cells were pretreated α-BTX (500 μM) 30 min before giving LPS insult and GSS treatment. M1 depolarization and NF-κB signaling activation was determined 24 h after GSS treatment. (A) Immunofluorescence results of IL-1β in BV2 cells (Scale bar = 500 μm, n=6). (B) ELISA results of IL-1β. (C-E) Western blot results of CD40 and CD68. (F) Immunofluorescence results of P65-NF-κB (Scale bar = 10 μm, n=6). (G-J) Western blot results of NF-κB signaling proteins.Comparisons between groups were carried out using one-way ANOVA followed by Newman-Keuls test. ^*^P<0.05.

Finally, we used α-BTX (an α7nAChR blocker) to determine whether the anti-M1 polarization effects of GSS was attributed to α7nAChR-mediated signaling. Both immunofluorescent staining and ELISA results showed that α-BTX pretreatment significantly reversed the effects of GSS on IL-1β protein expression in LPS-stimulated BV2 cells (Figure 6A and B). At the same time, α-BTX pretreatment restored the protein expressions of CD40 and CD68, which were significantly decreased by GSS treatment in LPS-stimulated BV2 cells (Figure 6C-E). GSS treatment significantly inhibited the nucleus translocation of P65-NF-κB in LPS-stimulated BV2 cells. Whereas, these effects of GSS were significantly attenuated by α-BTX pretreatment (Figure 6F). Western blot results also showed α-BTX pretreatment insignificantly blunted the inhibitive effects of GSS on NF-κB signaling. As shown in figure 6G-J, GSS treatment significantly reduced the levels of p-IKK and p-P65-NF-κB but increased IκB protein expression compared with LPS-stimulated group. In contrast, α-BTX pretreatment dramatically blocked these effects of GSS (Figure 6A-J).

## Discussion

In the present study, we found that GSS treatment significantly reduced brain infarcted volume and improved neurological function by inhibiting microglial M1 depolarization-mediated inflammation in tMCAO rats, with an underlying mechanism through upregulating α7nAChR expression and thereby blocking NF-κB signaling.

Our previous study showed that GSS treatment reduced infarcted brain area and improved neurological recovery in tMCAO rats and the mechanism underlying the therapeutic effects was through protecting against neuronal apoptosis 32. In that study, we found that GSS showed a dose-dependent effect, of which 1 mg/kg body weight had the best effect. Therefore, we adopted this dose in the present study. Besides, we also noticed that GSS treatment reduced MMP-3 and MMP-9 mRNA and protein expression 41, provoking us to investigate the anti-inflammation effects and its underlying molecular mechanism of GSS in ischemic stroke. Our results showed that GSS treatment reversed IL-1β expression in brain peri-ischemic regions and alleviated brain injury and neurological deficits in tMCAO rats. These results demonstrate that the GSS inhibits inflammatory responses in ischemic penumbra brain regions during ischemic stroke.

Neuroinflammation occurs rapidly after the onset of ischemic stroke and lasts for the whole disease process 42. It was reported that prolonged inflammation exacerbates brain injury and postpones brain function recovery 43. Microglia is one of the resident immune cells in the brain. Their activated states account for the dual effects of neuroinflammation in ischemic stroke: Microglial M2 phenotype is dominant in the early stages of ischemic stroke; it gradually switches to M1 phenotype 1. M2-depolarized microglia exert anti-inflammation effects by producing IL-10 and TGF-β. In contrast, M1-depolarized microglia is proinflammatory. Thereby, inhibiting microglia depolarization to M1 phenotype is considered as a potential strategy for the treatment of ischemic stroke 3. In this study, we used the microglial marker CD11b to determine the activation of microglia in the ischemic brain 44. Our results showed that microglia in ischemic penumbra regions was significantly activated, showing a rod-shaped morphology with a large cell body 44. In comparison, GSS treatment significantly inhibited the activation of microglia in ischemic penumbra regions where most microglia were ramified shape as showing in sham rats 45, 46. In this study, we used CD40 and CD68 further verified microglial M1 depolarization 47-49. Our results showed that CD40 and CD68 expression significantly increased in the petri-ischemic regions in tMCAO rats, while GSS treatment dramatically blocked the expression of these markers. Besides, we validated our findings through *in vitro* experiments: GSS treatment significantly blocked LPS-induced M1 depolarization and IL-1β expression in BV2 microglial cells. The M1 depolarized microglia are characterized by a set of markers (CD16, CD32, CD40, CD68 and CD86) and secrete proinflammatory cytokines such as IL-1β and TNF-α 50, 51. Hence, our results demonstrate that GSS treatment suppresses microglial M1 depolarization in tMCAO rats. Our previous study showed that GSS increased IL-10 expression in peri-infarcted regions in ischemic stroke rats (unpublished data), implying that GSS might promote microglia depolarization to M2 phenotype, which requires future research.

NF-κB, as a critical transcription factor, contributes to neuroinflammation through regulating microglia activation. In canonical NF-κB activation pathways, phosphorylated IKK protein promotes the degradation of IκB protein, thereby resulting in the release and subsequent nucleus translocation of P65-NF-κB protein, which regulates a variety of target genes expression 52. This study showed that GSS treatment significantly increased IκB protein expression but reduced the phosphorylation levels of IKK and P65-NF-κB protein in both tMCAO rats and LPS-stimulated BV2 cells. Moreover, GSS treatment also remarkedly inhibited the nucleus translocation of P65-NF-κB induced by LPS in BV2 cells. Our results suggest that GSS inhibits microglial M1 depolarization through blocking NF-κB signaling activation.

α7nAChR is a crucial receptor mediating cholinergic anti-inflammatory pathway. The stimulation of α7nAChR inhibits the activation of NF-κB signaling in microglia, reduced M1 microglia in the peri-infarct brain regions, and reduced brain injury in permanent MCAO (pMCAO) mice 2, 25. In this study, we found that α7nAChR protein expression significantly decreased in the ischemic penumbra regions in tMCAO rats, while it was restored by GSS treatment. Besides, GSS treatment also significantly inhibited the decrease of α7nAChR expression induced by LPS in BV2 cells. These results indicate that GSS upregulates microglial α7nAChR function in ischemic stroke. After pretreatment with α7nAChR inhibitor, α-BTX, we found that GSS lost the protection against brain infarction and neurological dysfunction and the inhibitive effects on microglial M1 depolarization and NF-κB signaling in both *in vivo* and *in vitro* experiments. These findings demonstrate that α7nAChR-mediated inhibition of NF-κB signaling is a vital molecular mechanism underlying the regulation of microglial M1 depolarization by GSS.

## Conclusions

**Figure 7:**
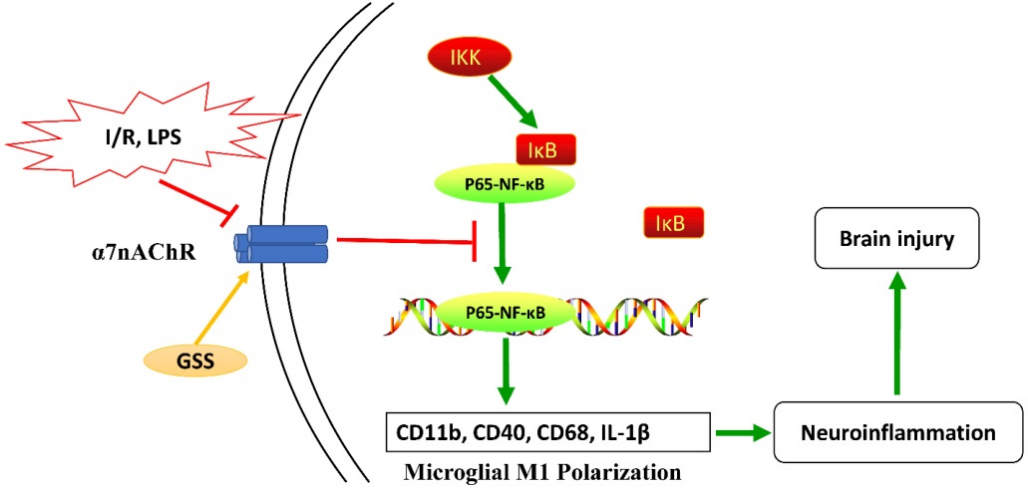
** Schematic diagram of the molecular mechanisms underly GSS against brain injury in tMCAO rats.** GSS treatment upregulates α7nAChR and thereby inhibits NF-κB signaling, subsequently suppresses M1 depolarization-mediated neuroinflammation in brain penumbra regions, protecting against brain injuries in tMCAO rats.

Taken together, as shown in Figure 7, our results from *in vivo* and *in vitro* experiments showed that GSS treatment upregulated α7nAChR and thereby inhibited the activation of NF-κB and microglial M1 depolarization, contributing to the neuroprotective effects in tMCAO rats. This study uncovers a novel molecular mechanism underlying the anti-ischemic stroke effects of GSS, demonstrating that GSS protected against brain ischemic injury by suppressing neuroinflammation via α7nAChR-mediated inhibition of NF-κB signaling.

## Supplementary Material

Supplementary figure S1.

## Figures and Tables

**Table 1:**
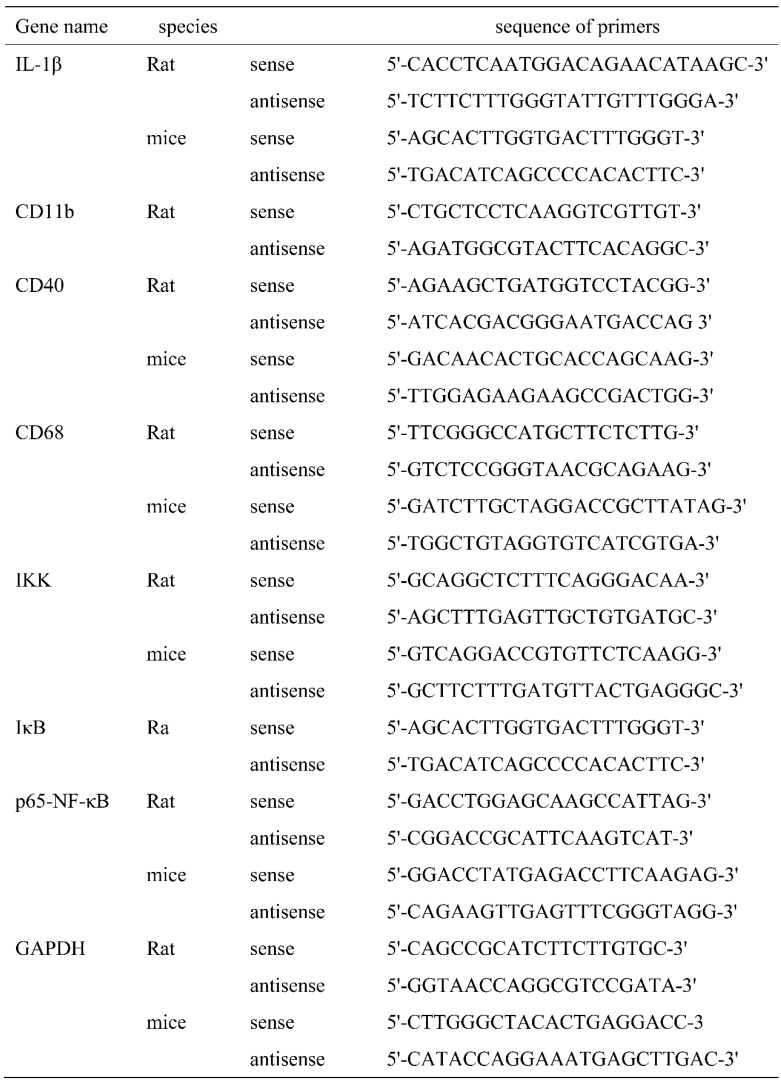
The sequence of primers.

## Abbreviations

ACh: acetylcholine
α7nAChR: α7 nicotinic acetylcholine receptor
α-BTX: α-bungarotoxin
ANOVA: analysis of variance
BBB: blood-brain barrier
CAIP: Cholinergic anti-inflammatory pathway
ELISA: enzyme-linked immunosorbent assay
GSS: Genistein-3′-sodium sulfonate
IL-1β: interleukin-1β
IL-6: interleukin-6
IL-10: interleukin-10
LPS: lipopolysaccharide
NF-κB: nuclear factor kappa B
SD: Sprague-Dawley
TGF-β: transforming growth factor β
tMCAO: transient middle cerebral artery occlusion
TNF-α: tumor necrosis factor α
tPA: tissue plasminogen activator
TTC: 2,3,5-Triphenyltetrazolium chloride
