# Supplementary material for: Genistein-3′-sodium sulfonate Attenuates Neuroinflammation in Stroke Rats by Down-Regulating Microglial M1 Polarization through α7nAChR-NF-κB Signaling Pathway
Source: Int J Biol Sci. 2021 Mar 8;17(4):1088–100. doi: 10.7150/ijbs.56800 (PMC8040300; doi:10.7150/ijbs.56800)
Supplement: Supplementary file 1 — Supplementary figure S1. [file ijbsv17p1088s1.pdf]

Supplementary Figure:

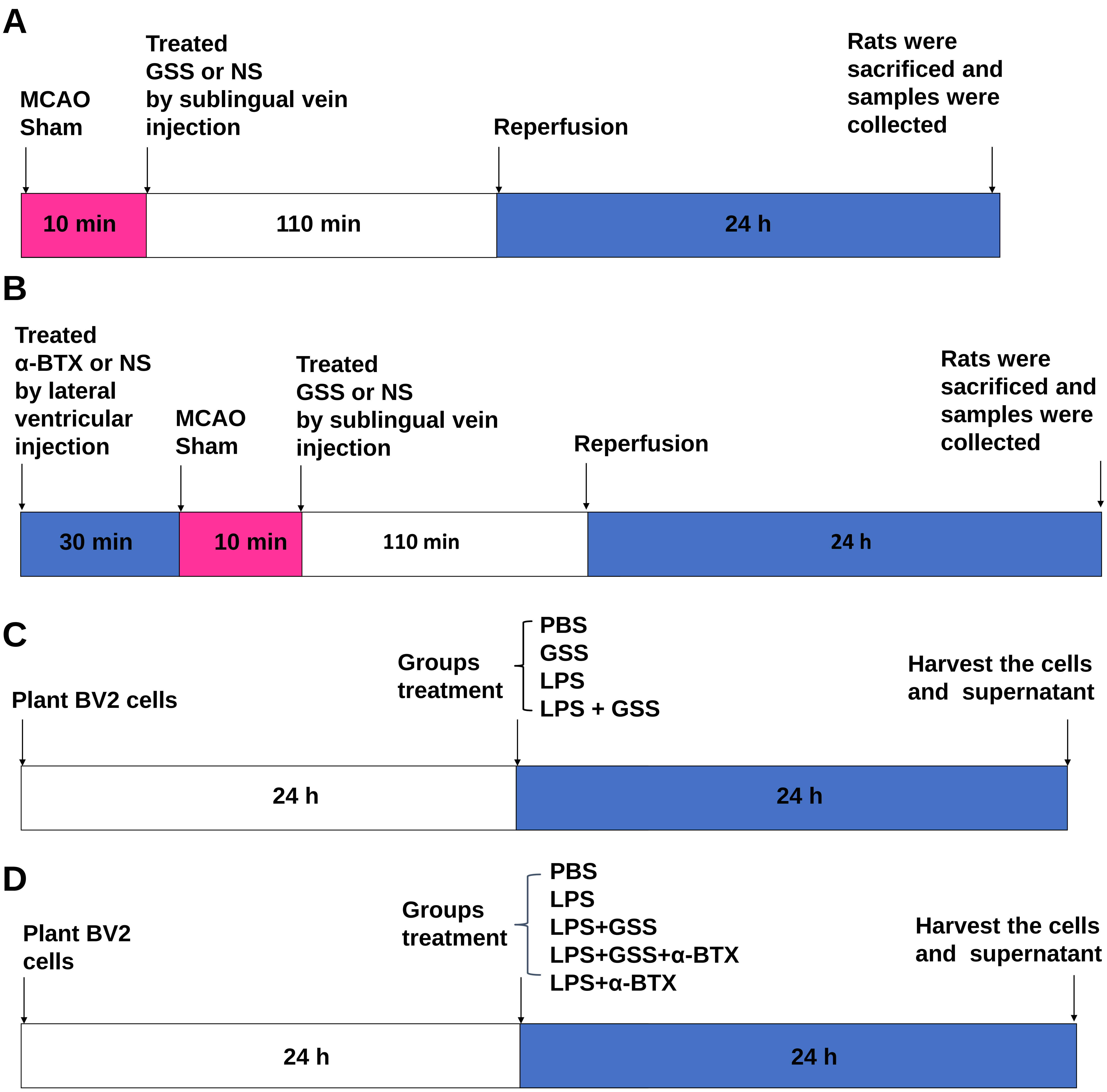

Figure S1 Schematic diagram for experimental scheme. A and B are the scheme for in vivo experiments. C and D are the scheme for in vitro experiments.
